# *In vitro* testing of a funnel-shaped tip catheter model to decrease clot migration during mechanical thrombectomy

**DOI:** 10.1038/s41598-019-57315-9

**Published:** 2020-01-20

**Authors:** Yasemin Tanyildizi, Emily Payne, Tiemo Gerber, Larissa Seidman, Axel Heimann, Oliver Kempski, Doris Leithner, Andreas Garcia-Bardon, Roman Kloeckner, Felix Hahn, Naureen Keric, Julia Masomi-Bornwasser, Marc A. Brockmann, Stefanie Kirschner

**Affiliations:** 1 University Medical Center Mainz, Department of Neuroradiology, Langenbeckstraße 1, 55131 Mainz, Germany; 2 University of Heidelberg, Medical Faculty Mannheim, Department of Radiation Oncology, Theodor-Kutzer-Ufer 1-3, 68167 Mannheim, Germany; 3 University Medical Center Mainz, Institute of Pathology, Langenbeckstraße 1, 55131 Mainz, Germany; 4 University Medical Center Mainz, Institute for Neurosurgical Pathophysiology, Langenbeckstraße 1, 55131 Mainz, Germany; 5 University Hospital Frankfurt, Department of Diagnostic and Interventional Radiology, Theodor Stern-Kai 7, 60590 Frankfurt, Germany; 6 University Medical Center Mainz, Department of Anesthesiology, Langenbeckstraße 1, 55131 Mainz, Germany; 7 University Medical Center Mainz, Department of Diagnostic and Interventional Radiology, Langenbeckstraße 1, 55131 Mainz, Germany; 8 University Medical Center Mainz, Institute for Neurosurgery, Langenbeckstraße 1, 55131 Mainz, Germany

**Keywords:** Stroke, Stroke

## Abstract

One limitation of mechanical thrombectomy (MT) is clot migration during procedure. This might be caused by abruption of the trapped thrombus at the distal access catheter (DAC) tip during stent-retriever retraction due to the cylindrical shaped tip of the DAC. Aiming to solve this problem, this study evaluates the proof-of-concept of a new designed funnel-shaped tip, in an experimental *in vitro* setting. Two catheter models, one with a funnel-shaped tip and one with a cylindrical-shaped tip, were compared in an experimental setup. For MT a self-made vessel model and thrombi generated from pig’s blood were used. MT was performed 20 times for each device using two different stent-retrievers, 10 times respectively. For the funnel-shaped model: for both stent-retrievers (Trevo XP ProVue 3/20 mm; Trevo XP ProVue 4/20 mm) MT was successful at first pass in 9/10 (90%), respectively. For the cylindrical-shaped model: MT was successful at first pass in 5/10 (50%) with the smaller stent-retriever and in 6/10 (60%) with the larger stent-retriever. The experiments show a better recanalization rate for funnel-shaped tips, than for cylindrical-shaped tips. These results are indicating a good feasibility for this new approach, thus the development of a prototype catheter seems reasonable.

## Introduction

Mechanical thrombectomy (MT) has become the standard technique in the treatment of acute ischemic stroke in large vessel occlusion (LVO). Multiple randomized controlled trials (RCTs) demonstrated a safe and efficient use of MT in comparison to intravenous tissue plasminogen activator (IV t-PA) use alone^1–6^.

Recently updated international and national guidelines^7,8^ recommend MT for patients suffering from acute stroke up to 24 hours after the onset of symptoms, as well as for patients with wake-up strokes with an unknown time window, thus emphasizing the increasing relevance of endovascular treatment. The so-called “big five”^1–5^ reports for MT in LVO show a good recanalization rate of 71%. A meta-analysis of 12 studies reports a successful recanalization rate (TICI 2b/3 score) of 81% for MT with stent- retriever in acute M2 occlusion^9^. Different techniques are possible for MT, like Direct Aspiration First Pass Technique (ADAPT), Stent-retriever Assisted Vacuum-locked Extraction (SAVE) or primary combined approach (PCA) of an aspiration catheter and stent-retriever^6,10^.

Even though MT in combination with IVT is the state-of-the-art treatment for LVO, there are still limitations. Balami *et al*.^11^ report an embolization to new vascular territories in 1–8.6% for most randomized controlled trials (RCTs) and in 1–12.5% for the non-RCTs. This can be caused by clot migration to proximal vessels during the retraction of the stent-retriever or persistence in the same vessel. Furthermore, the clot can break and dissipate in multiple smaller vessel branches during the procedure^12^. The risk of embolization to a new territory (ENT) increases during MT of a proximal clot^13^.

A different reason for clot migration or fragmentation might be the distal shape (tip shape) of the DAC, which is cylindrical in the commonly used DACs. Parts of the clot might abrupt at the DAC’s wall during retraction of the stent-retriever and dissipate into distal vessels, causing ENT. Even though flow arrest with an inflated balloon guide catheter (BGC) is frequently applied during MT^14^, a distal blood flow may remain, increasing the risk of dissipation of the broken clot with the blood flow into peripheral small branches.

To improve recanalization rates and to overcome the well-known limitations of endovascular MT, different alternative techniques for mechanical thrombectomy have been proposed and tested^6,10^. However, to the best of our knowledge no studies have investigated differently shaped tips of the DAC.

This study is evaluating the proof-of-concept of a funnel-shaped tip in an experimental *in-vitro* setting, using thrombi made from pig’s blood and a shortened intravenous (IV) line as a middle cerebral artery (MCA) model. Recanalization rates and clot migration were assessed and compared with MT results of a cylindrical-shaped tip.

This future catheter could either fit into commercially used, cylindrical shaped DACs such that the funnel shaped tip will be released at the distal end of the DAC as soon the catheter is outside of DAC’s lumen.

Or the catheter itself is used as a DAC and a flexible cover is used to hold the cylindrical shaped tip in place until the occluded vessel is reached. Once the occluded vessel is reached, the cover might be removed and the funnel shaped tip released.

## Materials and Methods

### Experimental setup

**Figure 1:**
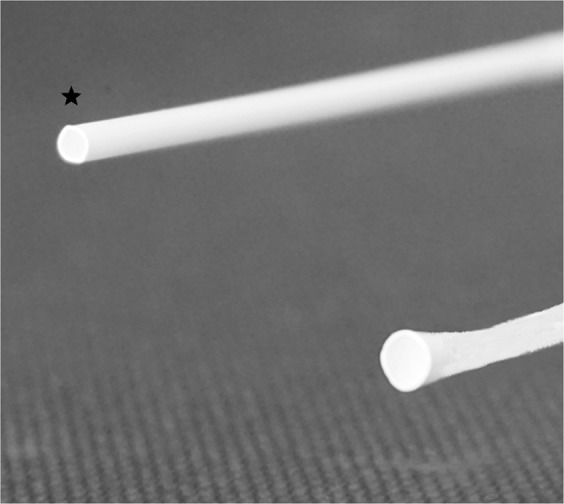
Standard cylindrical-shaped tip (asterisk) and modified funnel-shaped tip.

An experimental setup was used to investigate a funnel-shaped and a cylindrical-shaped tip for MT (Fig. 1).

The setup included two modified introducer sheaths (4 French (F) Radiofocus® Introducer II Standard Kit; TERUMO International Systems, Leuven, Belgium), which were used to simulate the tip of DACs. For this purpose the tapered tip of both introducer sheaths was removed, resulting in cylindrical-shaped introducer sheaths with a diameter of 1.5 mm. Afterwards, one of these models was manually dilated to a funnel-shape with a diameter of 2.5 mm. The cylindrical-shaped introducer sheath with 1.5 mm diameter was used a standard model, simulating a commonly used DAC. The funnel-shaped introducer sheath was used as a novel model, simulating a modified (not yet existing) DAC.

The IV line of a blood transfusion kit (CODAN, Medizinische Geräte GmbH & Co KG, Lensahn, Germany) with an inner tube diameter of 3.0 mm was used to function as MCA. The tube was shortened to a length of 16 cm.

A shortened (3 cm) 9F introducer (Radiofocus® Introducer II Standard Kit; TERUMO International Systems, Leuven, Belgium) was used to access the MCA model, so as to bring in the material for the interventional procedure.

As human blood substitute, a transparent non-Newtonian aqueous glycerol solution was used at room temperature. To mimic a blood like viscosity of the fluid, distilled water and 40% glycerol (Glycerin 85%; Caesar & Lorenz GmbH, Hilden, Germany) were freshly mixed prior to each experiment^15,16^.

### Processing of thrombi

A Chandler Loop System enabling the simulation of extracorporal blood circulation was used to generate thrombi. Venous blood was taken from pigs (German Landrace) used in approved final animal experiments prior to being sacrificed.

Immediately after taking blood, polyvinyl chloride (PVC) laboratory tubes (clear PVC tubing, inner diameter 8.0 mm, outer diameter 12.0 mm; Thermo Scientific Fischer, Waltham, Massachusetts, USA) were half filled with blood, closed and transferred to the rotation unit of the Chandler Loop System. The rotation unit was set to 15 rotations per minute and the temperature controlled water basin was preheated to 38.5 °C. After the blood had clotted (approximately after 20 minutes), the tubes were removed, the thrombi captured in sodium chloride and stored in a refrigerator for a maximum of two days until used in the experiment.

#### Ethics approval

Animal experiments were carried out after receiving approval by the local governmental committee (Landesuntersuchungsamt Rheinland-Pfalz, Germany) under the reference number 23 177-07/G 14-1-094 and in accordance with the German Animal Welfare Act (Tierschutzgesetz). All applicable international, national and/or institutional guidelines for the care and use of animals were adhered to.

### Thrombectomy procedure

For thrombectomy procedure thrombi were cut in 20 mm pieces. Under aspiration a thrombus was brought into the MCA model that had already been flushed with a blood substitute. After the thrombus had been placed in the right position the system was closed and one of the two different models was inserted into the MAC model. Then a stent-retriever (Trevo® XP ProVue 3/20 mm and Trevo® XP ProVue 4/20 mm; Stryker, Kalamazoo, Michigan, USA) was introduced and released when the side of the blood clot was reached.

**Figure 2:**
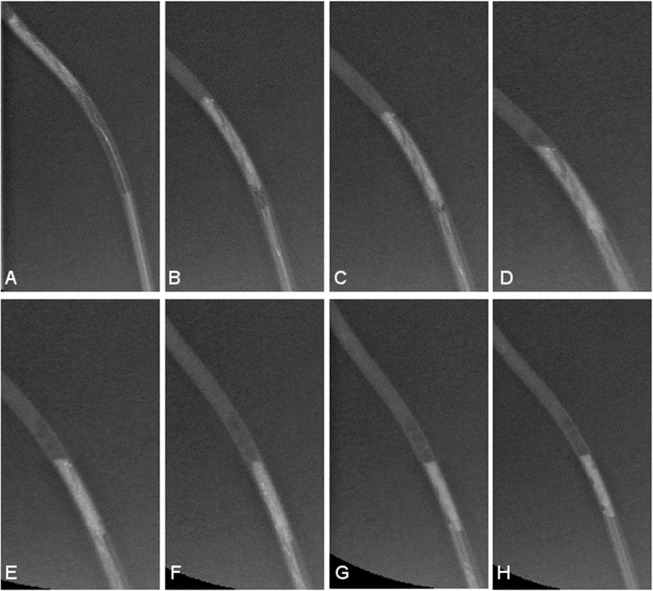
Cylindrical-shaped tip during mechanical thrombectomy (**A–H**) showing abruption of the thrombus at the wall of the catheter wall (**D–H**), while retracting the trapped thrombus into the model.

**Figure 3:**
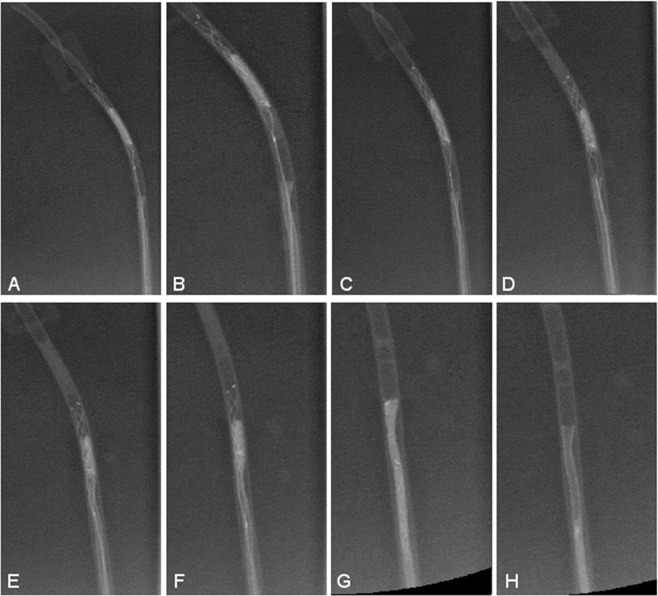
Funnel-shaped tip during mechanical thrombectomy (**A**–**H**) without thrombus loss. (**A**) and (**B**) are the same images with (**B**) zoomed in, showing the funnel-shaped tip.

Subsequently, the stent-retriever was deployed for three minutes and then pulled back into the standard or novel model under aspiration with a vacuum pressure syringe (60 ml, VacLok®, Vacuum pressure syringes; Merit Medical System 60 ml) set to a negative pressure vacuum of 40 mmHg (Figs. 2 and 3, see also supplementary information).

### Histopathological analyses

After immersion fixation with 4% buffered formalin, the thrombi were sectioned at 3 mm intervals. Thrombi sections were doubly embedded. First, they were placed in agar for proper orientation and then embedded in paraffin, cut at 2 µm thickness onto slides and stained with Masson trichrome.

All samples were scanned with Nanozoomer 2.0 HT (Hamamatsu Photonics K.K., Hamamatsu, Japan) with a 200 × magnification. The images were exported as TIFF file to determine the exact pattern of fibrin distribution. First, the Trainable Weka Segmentation Fiji plugin v3.2.27^17^ was used to perform a pixel-by-pixel analysis of the scanned images for automatically differentiating fibrin and thrombus. To accomplish this, a training set of ten pictures cropped to 300 × 300 pixels was created and three classifiers were marked: fibrin, thrombus, and background. The classifier training process was repeated using different image filter combinations. The best result was generated using a Hessian matrix filter in combination with a mean voxel distance calculation. Detailed machine learning algorithms to produce pixel based segmentations are available online^18,19^. Images were further processed and transformed into grayscale images. Then a binary image with segmented particles was generated. Two pathologists blinded to the sample groups executed the final step, which was graduating each thrombus in the categories pattern, localization and distribution.

### Statistical analyses

Statistical analyses were performed using SPSS Software (23.0) (IBM, New York, USA). The level of significance was calculated by means of the chi-square-test (Χ² test). The level of significance was set at α = 0.05.

## Results

Mechanical thrombectomy was performed 20 times for each tip, using two different stent-retrievers, 10 times respectively.

### Results for the funnel-shaped tip

MT was successful at first pass 9 out of 10 times (90%), respectively, for the funnel-shaped model, using a Trevo XP ProVue 3/20 mm and a Trevo XP ProVue 4/2. For both stent-retrievers, MT failed 1 time due to thrombus loss during stent-retriever retraction, before reaching the tip (Table 1).

**Table 1:** Results of the mechanical thrombectomy with the funnel-shaped and cylindrical-shaped models using two different stent-retrievers: Trevo XP ProVue 3/20 mm and Trevo XP ProVue 4/20 mm.

Stent retriever type	Funnel-shaped tip	Cylindrical-shaped tip	p-value
Trevo XP ProVue 3/20 mm (n = 10) **positive mechanical thrombectomy**	9	5	0.05
Trevo XP ProVue 4/20 mm (n = 10) **positive mechanical thrombectomy**	9	6	0.12

### Results for the cylindrical-shaped tip

For the cylindrical-shaped tip 5 out of 10 (50%) MTs were successful during first pass with the Trevo XP ProVue 3/20 mm. MT was not successful in all 4 times due to abruption of the clot at the wall of the tip, 1 time due to thrombus loss during stent-retriever retraction before reaching the model. With the Trevo XP ProVue 4/20 mm, 6 out of 10 (60%) MTs were successful at first pass. In 2 cases the thrombus was lost due to abruption at the wall of the tip, in the other 2 cases due thrombus loss during stent-retriever retraction before reaching the model (Table 1).

### Results for the histopathological analyses of the thrombi

**Figure 4:**
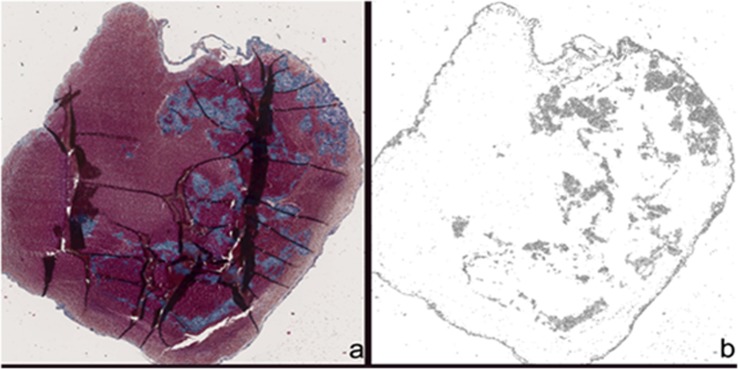
Thrombus before (**a**) and after (**b**) segmentation calculation; the histopathological analysis showed a reticulated pattern for the fibrin.

Above all, the thrombi showed a different pattern, 48 out of 134 (36%) were reticulated (Fig. 4). Further graduation of each thrombus in the categories patter, distribution and localization is depicted in Table 2.

**Table 2:** Histopathological analysis of the thrombus with graduation of each thrombus in the categories distribution, pattern and localization.

Distribution	Frequency	Pattern	Frequency	Localization	Frequency
Irregular	103 (76.8%)	Insular	52 (38.8%)	Central and Peripheral	51 (38.1%)
Regular	31 (23.2%)	Reticular	48 (35.8%)		
		Patternless	34 (25.3%)	Peripheral	29 (21.6%)
				Central	7 (5.2%)
				Patternless	47 (35.1%)

## Discussion

MT in combination with intravenous thrombolysis (IVT) is the recommended treatment for acute large vessel occlusion^20,21^. One limitation of MT is clot fragmentation and migration.Our approach to improve MT at first pass and to hinder clot fragmentation is to modify the established cylindrical-shaped tip of DACs into a self-expanding funnel-shaped tip.

The development of a prototype is cost-intensive and preliminary research results are needed to find a partner interested in the idea and to support further product development. Therefore, an experimental *in vitro* set-up was used for proof- of principle, hereby respecting and reflecting the diameters of a real catheter and cerebral blood vessels. The inner diameter of a standard DAC (e.g. SOFIA®, MicroVention Inc. Aliso Viejo, California, USA, with 5F (ID 1.39 mm (0.055 inch) or 6F 1.78 mm (0.070 inch), is reflected by the used model (cylindrical tip: 1.5 mm). Average diameter of the MCA is reported with 2.5–3 mm in the literature^22^, this was reflected by the study concept (funnel shape with 2.5 mm and silicon tube (simulating a vessel) with 3 mm diameter.

Commercially available 3D silicon MCA models could not be used since vessel lengths of anatomical models distinctly exceed the length of the introducer sheaths used for MT. In comparison to a standard, cylindrical-shaped tip, the novel funnel-shaped tip showed promising advantages in the study at hand. We observed a 90% recanalization rate at first-pass, showing no abruption of the clot at the model wall, regardless of the stent-retriever size. MT failed two times, however not due to abruption of the clot but rather to a thrombus loss during stent retraction before reaching the tip. In our *in vitro* setting we could observe that the thrombus was not trapped sufficiently in the stent-retriever. This might happen during real MT as well. The cylindrical-shaped tip showed lower recanalization rates, with 50% for the smaller stent-retriever size (3/20 mm) and 60% for the larger stent-retriever size (4/20 mm). In four, respectively in two cases, the clot was lost due to abruption at the DAC wall. We were able to observe that in larger stent-retrievers the number of exceeding parts of the clot was less than in smaller stent-retrievers. This might change depending on the size of the clot. Since the clot size in the study at hand was standardized to 20.0 mm (length) × 0.8 mm (diameter), an increased overlap of clot for the smaller stent-retriever was observed leading to higher potentials of clot abruption, which is emphasized by our results (4 times abruption of the clot with the 3/20 mm stent- retriever vs. 2 times with the 4/20 mm).

The catheter model used in this study was not tested in terms of navigation. Thinking of a funnel-shaped tip, it might be difficult and dangerous to reach the target vessel. Two options to reach the cerebral vessel might be possible. Firstly a thin cover (like a guide catheter) could be an option to hold the self expanding funnel shaped tip in place, until the target vessel is reached. After the catheter has been positioned, the cover could be retracted and the funnel shape could be released, hereby occluding the vessel. The self-expanding power ensures a flexible shape, which can adapt to various vessel diameters, hereby sealing the vessel lumen and preventing clot fragments from stripping off during retraction of the stent-retriever. The funnel-shaped tip will be foldable, using the guide catheter, if the DAC has to be repositioned. This ensures a save transportation to even distal blood vessels with small lumen without damaging the vessel walls. Alternatively the commonly used DAC might be the guiding catheter for the self-expanding funnel shaped tip. After the stent retriever is released, the funnel shaped tip catheter could reach the target vessel, through the standard DAC and when the DAC is removed, the funnel shape will be released. With this no navigation would be necessary and the normal DAC itself could be used as a sheath. Both options might be assessed, when a prototype catheter is eligible for *in vitro* and *in vivo* experiments.

The application of a funnel-shaped aspiration catheter may also be beneficial during acute thromboembolic occlusion of vessels other than cerebral arterial vessels, like mesenteric arteries. Commonly, no stent-retriever is used during endovascular recanalization in acute mesenterial ischemia; the clot is removed through aspiration with a so called aspiration catheter, only^23^. The use of an aspiration catheter with a self-expanding funnel-shaped tip would occlude the vessel, enabling easier aspiration and diminishing the risk of clot migration. This hypothesis has not been evaluated yet and has to be tested in *in vitro* and *in vivo* settings with a prototype.

A general limitation of the study is the single *in vitro* experimental setting, with missing *in vivo* experiments. The reason for this is the not yet feasible prototype of the novel DAC for *in vivo* experiments. To show the safety and the efficiency of our proposed modification and before using the new funnel-shaped tip DAC in humans, further *in vitro* and *in vivo* animal studies are needed.

Additionally, only one clot type was used, with high proportion of fibrin. Since different clot types and clot sizes may have an influence on recanalization rates and clot migration, different clot types have to be included in further studies. Apart from that, in patients with acute ischemic LVO recombinant tissue plasminogen activator (rtPA) is applied intravenously before endovascular treatment, which can change clot character. The influence of rtPA during MT using a funnel shaped DAC, has to be evaluated in future studies.

The limited number of attempts and the evaluation of only one MT technique (PCA), limited this study. A restricted amount of pig’s blood available allowed only the production of a small amount of thrombi. Therefore, we decided to evaluate only one the combined MT technique.

However, our very first study design and results encourage us to develop a prototype with a self-expanding funnel-shaped tip and perform more *in vitro* and *in vivo* animal studies.

## Conclusion

To decrease clot migration during MT a new approach with a funnel-shaped catheter tip was suggested. The underlying proof of principle study shows a better recanalization rate for funnel-shaped tips than for cylindrical-shaped tips. These results are indicating a good feasibility for this new approach, thus the development of a prototype catheter seems reasonable. Further *in vitro* and *in vivo* studies with prototype catheter are needed to evaluate the efficacy of the novel DAC, hereby assessing recanalization rates and clot migration.

## Supplementary information

Video 1.

Video 2.
